# To the Future: The Role of Exosome-Derived microRNAs as Markers, Mediators, and Therapies for Endothelial Dysfunction in Type 2 Diabetes Mellitus

**DOI:** 10.1155/2022/5126968

**Published:** 2022-02-21

**Authors:** Maurice B. Fluitt, Neal Mohit, Kanwal K. Gambhir, Gail Nunlee-Bland

**Affiliations:** ^1^Division of Endocrinology and Metabolism, Department of Medicine, Howard University College of Medicine, 520 W St NW, Washington, DC 20059, USA; ^2^Department of Biology, Howard University, 415 College St. NW, Washington, DC 20059, USA; ^3^Diabetes Treatment Center, Howard University Hospital, 2041 Georgia Ave, NW, Washington, DC 20060, USA

## Abstract

The prevalence of diabetes mellitus (DM) is increasing at a staggering rate around the world. In the United States, more than 30.3 million Americans have DM. Type 2 diabetes mellitus (T2DM) accounts for 91.2% of diabetic cases and disproportionately affects African Americans and Hispanics. T2DM is a major risk factor for cardiovascular disease (CVD) and is the leading cause of morbidity and mortality among diabetic patients. While significant advances in T2DM treatment have been made, intensive glucose control has failed to reduce the development of macro and microvascular related deaths in this group. This highlights the need to further elucidate the underlying molecular mechanisms contributing to CVD in the setting of T2DM. Endothelial dysfunction (ED) plays an important role in the development of diabetes-induced vascular complications, including CVD and diabetic nephropathy (DN). Thus, the endothelium provides a lucrative means to investigate the molecular events involved in the development of vascular complications associated with T2DM. microRNAs (miRNA) participate in numerous cellular responses, including mediating messages in vascular homeostasis. Exosomes are small extracellular vesicles (40-160 nanometers) that are abundant in circulation and can deliver various molecules, including miRNAs, from donor to recipient cells to facilitate cell-to-cell communication. Endothelial cells are in constant contact with exosomes (and exosomal content) that can induce a functional response. This review discusses the modulatory role of exosomal miRNAs and proteins in diabetes-induced endothelial dysfunction, highlighting the significance of miRNAs as markers, mediators, and potential therapeutic interventions to ameliorate ED in this patient group.

## 1. Introduction

Diabetes mellitus is a growing epidemic that poses a major global health threat [1]. The 10^th^ edition of the International Diabetes Federation (IDF) Diabetes Atlas estimates that 527 million people have diabetes [1]. It is the seventh leading cause of death and continues to rise at an alarming rate in the United States and abroad. In the United States, an estimated 34.2 million Americans have diabetes mellitus. While Type 1 Diabetes Mellitus (T1DM) represents about 5% of the diagnosed cases, Type 2 Diabetes Mellitus (T2DM) accounts for the majority of cases (90-95%) and has greatly contributed to the burden of mortality and disability of this complex disease [2]. In addition, the alarming increase of T2DM in the United States and abroad has placed a significant burden on healthcare systems, government officials, families, and caretakers, as the cost associated with the treatment and management of T2DM and its multisystem complications continue to rise. The prevalence of T2DM is disproportionally higher in minority populations such as Hispanics and African Americans. In fact, African Americans are twice as likely to be diagnosed with diabetes. Additionally, African Americans have two to four times the rates of T2DM-associated complications than European Americans [3].

The complex molecular underpinnings of T2DM and its complications consist of an interactive matrix of genetic, epigenetic, and lifestyle factors operating in a physical-sociocultural environment [2] . Specifically, T2DM is traditionally thought to develop during overconsumption of energy dense foods and lack of physical activity that conspires with genetic susceptibility, ultimately disrupting feedback loops between insulin action and secretion [4]. This disruption can result in impaired insulin signaling, *β*-cell function, and glucose metabolism and lead to persistent hyperglycemia. Both *β*-cell dysfunction and insulin resistance occur early in the pathogenesis of T2DM and over time drive the progression of normal glucose tolerance (NGT) to impaired glucose tolerance (IGT) to T2DM [2]. Additionally, other factors influencing the development of T2DM include population aging, intrauterine environment, changes in the microbiome, and pollution.

microRNAs (miRNAs) are a class of noncoding RNAs that function to fine tune gene expression of about 60% of protein-coding genes, including genes associated with inflammation, oxidative stress, and hemodynamic response. Dysregulated and altered miRNA expression has been implicated in the pathogenesis of T2DM and its complications, including ED. Previous work reveals that miRNAs are crucial regulators of endothelial cell function via modulation of eNOS-derived nitric oxide (NO) bioavailability, angiogenesis, and innate immune response [5]. The presence of miRNAs in both tissue and circulation (free and packaged in extracellular vesicles, i.e.; exosomes) makes miRNAs attractive biomarkers, mediators, and therapies for T2DM and its complications [6]. This review focuses on the role of exosomal miRNAs in diabetes-induced endothelial dysfunction, highlighting the significance of exosomal miRNAs as markers, mediators, and potential therapeutic interventions.

## 2. miRNAs in T2DM and Diabetic Vascular Complications

The vascular endothelium is a highly active endocrine organ involved in the regulation of vascular tone and vascular homeostasis. It secretes vasoactive molecules that act in an autocrine, paracrine, or endocrine manner, mediating the links between vascular function and metabolic demands [7, 8]. The secretion of vasoactive molecules regulates important physiological outcomes including vessel tone, diameter, platelet activation, leukocyte activation, and vascular smooth muscle cell proliferation (VSMC) [8]. Thus, dysfunction here results in a series of macro and microvascular complications. This dysfunction is often exacerbated in the complex setting of type 2 diabetes mellitus.

ED is a common finding in type 2 diabetic patients and increases the risk of incident cardiovascular events in this patient group. In T2DM, endothelial function is compromised by a series of metabolic derangements from insulin resistance and hyperinsulinemia to oxidative stress and excess free fatty acid release [7, 9, 10]. These persistent metabolic events negatively impact endothelial function and trigger a series of proatherogenic and inflammatory events, ultimately increasing the risk of macro and microvascular events—including cardiovascular disease (CVD) and diabetic nephropathy (DN) in this patient group. Thus, the endothelium provides a lucrative means to investigate the molecular events in the development and progression of cardiovascular and renal complications in T2DM.

There is a delay from the onset of T2DM and clinical diagnosis, resulting in chronic vascular complications [11–13]. In fact, newly diagnosed T2DM patients present with at least one vascular complication at diagnosis [11, 14], highlighting the lasting negative impact of high glucose and other diabetogenic agents (i.e., advanced glycated end-products, transforming growth factor-B, reactive oxygen species, and angiotensin II) on vascular endothelial cells. These negative insults persist even after “normalizing” glucose levels and compromise endothelial function due to the persistent long-term expression of DN-related genes, which undergo some form of epigenetic modification. Thus, some individuals with diabetes present with a continued progression of vascular dysfunction, even after glycemic control, due to a unique phenomenon known as metabolic memory [15, 16].

microRNAs can affect the function of vascular cells as key regulators of important cellular processes in endothelial cells, including cell migration, proliferation, angiogenesis, and inflammatory and oxidative responses [17–19]. Previous studies have confirmed that dysregulated and altered miRNAs are involved in endothelial homeostasis in diabetes [20–23]. For example, the endothelial cell-specific miR-126 promotes angiogenesis in response to angiogenic growth factors [21]. Wang et al. showed that miR-126 modulates angiogenesis by targeted deletion of miR-126 in mice [22]. In this study, the deletion of miR-126 resulted in vascular leakage and hemorrhaging [22]. These abnormalities were attributed to reduced angiogenic growth factor signaling, which resulted in reduced endothelial cell growth, sprouting, and adhesion [22]. In addition, Zheng et al. reported that serum miR-126 is reduced in patients with diabetic retinopathy [23]. They conclude that miR-126 targets PLK4 to reduce diabetic retinopathy by suppressing endothelial cell proliferation and migration in male Sprague-Dawley rats with streptozotocin- (STZ-) induced diabetes and in vitro in human retinal capillary endothelial cells [23]. MiR-34a, which promotes senescence in the vascular endothelium, is also a reported mediator of vascular pathologies in diabetes mellitus. In the diabetic vasculature, endothelial miR-34a is upregulated via recruitment of the p66Shc adaptor protein (a regulator of cellular redox state and apoptosis) [24] and targets Sirt1 to drive endothelial dysfunction under diabetic conditions [25]. Collectively, these studies highlight the important and diverse role of miRs in endothelial function and provide attractive avenues to investigate and develop potential therapies to ameliorate endothelial dysfunction in diabetic patients.

## 3. Extracellular miRNAs in Type 2 Diabetes

miRNAs detected in biological fluids (blood, urine, saliva, tears, and breastmilk) serve as ideal biomarkers for disease, including type 2 diabetes and its associated endothelial dysfunction [6]. There are several important characteristics represented by good biomarkers. These important characteristics or features include specificity, sensitivity, stability, noninvasiveness, robustness, and reproducibility [26, 27]. Previous studies suggest the use of circulating microRNAs as sensitive, specific, and noninvasive markers for early detection and monitoring of diabetes mellitus (type 1 and type 2) and its micro and macrovascular complications [6, 20, 26–33]. A study conducted by Zampetaki et al. provided the initial evidence for a prognostic plasma miRNA signature in diabetic patients [20]. They identified 11 altered plasma miRNAs associated with diabetes mellitus, including miR-126, which was reportedly lower at baseline in normoglycemic participants who later developed DM over the 10-year follow-up period [20]. These findings highlight the predictive power of miR-126 to identify study participants who were at risk for developing diabetes in later life. Early studies from our group also suggest the use of circulating miRNAs as biomarkers of type 2 diabetes in African American adults [6]. We identified 3 altered miRNAs (miR-15a, miR-15b, and miR-499) in prediabetic adults [34]. Additionally, Kamalden and colleagues also reported an increase in plasma miR-15a in diabetic patients, corroborating our early findings [34]. In a study investigating islet-specific miR-7, researchers found that serum miR-7 was significantly higher in type 2 diabetes and type 2 diabetes with associated microvascular complications [35]. In a cohort of type 2 diabetes with and without diabetic retinopathy (a common microvascular complication of type 2 diabetes), investigators recently reported that diabetic retinopathy is associated with higher plasma miR-25-3p and miR-320b [36]. Conversely, miR-495-3p was reportedly lower in patients with diabetic retinopathy [36]. miR-210 is also associated with diabetes mellitus and diabetic retinopathy, reportedly by mediating functional responses in endothelial cell function [37]. In a study of 150 type 2 diabetic patients, Yin et al. found that serum miR-210 was increased in patients with diabetic retinopathy and was positively correlated with HbA1c, HOMA-IR, and fasting plasma glucose [37]. They also report that miR-210 could differentiate patients with diabetic retinopathy from the healthy controls, highlighting the diagnostic potential of this miR [37]. Conversely, it was recently reported that miR-210 is lower in human RBCs in T2DM patients [38]. In endothelial colony-forming cells isolated from peripheral blood of diabetic patients, Luo et al. found an increased expression of miR-139-5p [35]. The findings of these studies support the use of circulating miRNAs as markers of disease and suggest that circulating miRs may be useful prognostic tools in diabetes mellitus and its complications (Table 1).

Since these early reports on circulating miRNAs as noninvasive markers of disease, researchers have made meaningful strides in understanding how and why extracellular miRNAs are exported from cells in physiological and pathophysiological states, especially during the diabetic milieu. There is sufficient evidence revealing that circulating miRNAs are protected from RNAse degradation by being packaged into small vesicles as cargo [6, 20, 39]. However, additional studies are still necessary to fully understand both how and why miRNAs are exported from cells. One sophisticated way miRNAs are transported out of the cell involves trafficking of membrane vesicles, which was initially described by Valadi et al. in 2007 [40].

## 4. Classification of Exosomes

The release of membrane vesicles is highly conserved in both prokaryotes and eukaryotes, highlighting the importance of these extracellular vesicles (EVs). All cells release EVs under both physiological and pathophysiological conditions [41]. Although the classification of EVs continues to evolve, EVs can be classified into two broad categories—ectosomes and exosomes—based on the generation and release of the vesicle. Ectosomes, for example, are generated by the outward budding of the plasma membrane and produce macrovesicles, microparticles, and larger vesicles that range in size from 50 nm to 1 *u*m in diameter [41]. Conversely, exosomes are small membrane (40-160 nm; ~100 nm on average) vesicles of endocytic origin [41]. Both play important roles in cell-to-cell communication through the horizontal transfer of cellular cargo, such as DNA, proteins, mRNA, and miRNAs [41, 42].

Exosomes are generated from the late endosome as part of the endosomal pathway [43]. During invagination of the late or maturing endosomal membranes, the mature endosome becomes a multivesicular body. The endosome or multivesicular body (MVB) then forms an intraluminal vesicle after the outer membrane of the MVB inverts itself, creating a “right side-out” membrane orientation, in which certain cytosolic proteins are incorporated into the invaginating membrane, and cytosolic components are enclosed in the intraluminal vesicles [43, 44]. The MVB can then either (1) be shuttled to the endolysosomal degradative pathway and fuse with a lysosome to under degradation and recycling of its contents or (2) be routed to the cell surface to be released from various cell types into extracellular space after fusion with the plasma membrane forming an exosome [43–45]. Exosomes often mirror the content of the parent cell, containing a large variety of constitutive elements [44]. These include a host of lipids, proteins, mRNAs, miRNAs, and long noncoding RNAs, highlighting the functional diversity and potential of exosomes [44–47]. Once released into the extracellular space from its parent cell, exosomes and exosomal content like protein, mRNAs, miRNAs, and other noncoding RNAs can be taken up by neighboring or distant cells to mediate a functional response in these recipient cells [48–50]. Thus, exosomes have emerged as novel markers of disease and mechanisms of cell-to-cell communication.

## 5. Exosomal miRNAs as Markers of Endothelial Dysfunction

Detection of exosomes in biological fluids such as blood, saliva, urine, tears, and breastmilk makes exosomes attractive noninvasive biomarkers of disease. Exosomes can reflect its cellular origin and physiological conditions as a fingerprint of the donor cell [44]. Therefore, the varying levels of exosomes and its rich exosomal cargo—like proteins, mRNAs, and miRNAs— prior to and during physiological and pathophysiological state can provide useful prognostic and diagnostic markers of disease. In addition, it may also provide insight into the complex cellular processes of diseases, like type 2 diabetes and the underlying endothelial dysfunction driving the vascular complications associated with this metabolic disease, like DN.

In a recent report, Xiong et al. found that miR-20b-5p was higher in exosomes isolated from patients with type 2 diabetes mellitus [51]. Katayama et al. also reported that serum-derived exosome-enriched circulating exosomal miR-20b-5p was significantly higher in type 2 diabetic men, compared to the healthy controls [52]. Additionally, a study investigating the effects of exosomes derived from mouse brain endothelial cells as treatment for stroke in type 2 diabetic mice found a significant decrease in serum and brain tissue miR-126 in the type 2 diabetic stroke (T2DM-stroke) mice when compared to the non-DM stroke mice [53]. The investigators of this study report that both endothelial cell and exosomes derived from mouse brain endothelial cells contain high levels of miR-126, in comparison to exosomes derived from smooth muscle cells, marrow stromal cells, and astrocytes [53].

Perhaps where exosomal miRNAs have the greatest use as early markers of the development and progression of diabetic complications is diabetic nephropathy (DN). DN is one of the most common and severe diabetic complications and is the leading cause of end-stage renal disease in diabetics. DN is characterized by ultrastructural, morphological, and functional changes that result from poor glycemic control and elevated blood pressure [54]. Thus, identifying and validating exosomal miRNAs (i.e., urinary exosomal miRNAs) as early markers of DN is necessary in identifying and understanding the events that occur well before the clinical manifestation of elevated proteinuria and declining eGFR. Previous studies have highlighted the use of urinary exosomal miRNAs as markers and predictors of renal function in both type 1 and type 2 diabetes [28, 29, 31, 33, 37, 55–60]. In one study, investigators profiled urinary exosomal miRNAs in a cohort of healthy and type 2 diabetic patients with and without diabetic nephropathy (a common microvascular complication of type 1 and type 2 diabetes) and identified 16 altered miRNAs, most of which were involved in the progression of renal diseases [28]. Interestingly, most of the deregulated urinary exosomal miRNAs were found in patients with microalbuminuria [28]. Of the 16 urinary exosomal miRNAs identified in this study, two miRNAs—miR-320c and miR-6068—were higher in the DN group [28]. Eissa et. al also identified several urinary exosomal miRNAs in the DN patients [61]. They identified elevated levels of urinary exosomal miR-133b, miR-342, and miR-30a in the T2DM DN patients in comparison to the healthy normal controls [61]. In another study investigating urinary exosomal (UE) miRNAs as markers of chronic kidney disease conducted by Kumari et al., they found that UE miR-451 was higher in subjects with early-stage chronic kidney disease [60]. Recently, Kumari et al. found that urinary exosomal miR-451 was approximately two-fold higher in the subjects with early chronic kidney disease [33]. Previous work from this group reports similar findings in diabetic rats, reporting an elevated excretion of urinary exosomal miR-451 in the diabetic group [33], highlighting the translational utility of this miRNA as an early marker of DN. In addition, the rise in urinary exosomal miR-451 could predict albuminuria in diabetic rats [33]. In another study profiling urinary extracellular vesicles, Prabu and colleagues identified 73 miRNAs. Of the 73 miRNAs, they found a unique urinary extracellular vesicle miRNA signature comprised of elevated levels of let-7i-3p, miR-24-3p, and miR-27b-3p, while levels of miR-15b-5p were decreased in type 2 diabetic patients with microalbuminuria [62]. This signature was successful at identifying patients with microalbuminuria in type 2 diabetes with normal urine albumin [62]. Taken together, these findings provide foundational support for the use of exosomal miRNAs as markers of endothelial dysfunction (Table 2) underlying the macro and microvascular complications, such as diabetic nephropathy, associated with T2DM.

## 6. Exosomal miRNAs as Mediators of Endothelial Dysfunction

**Figure 1:**
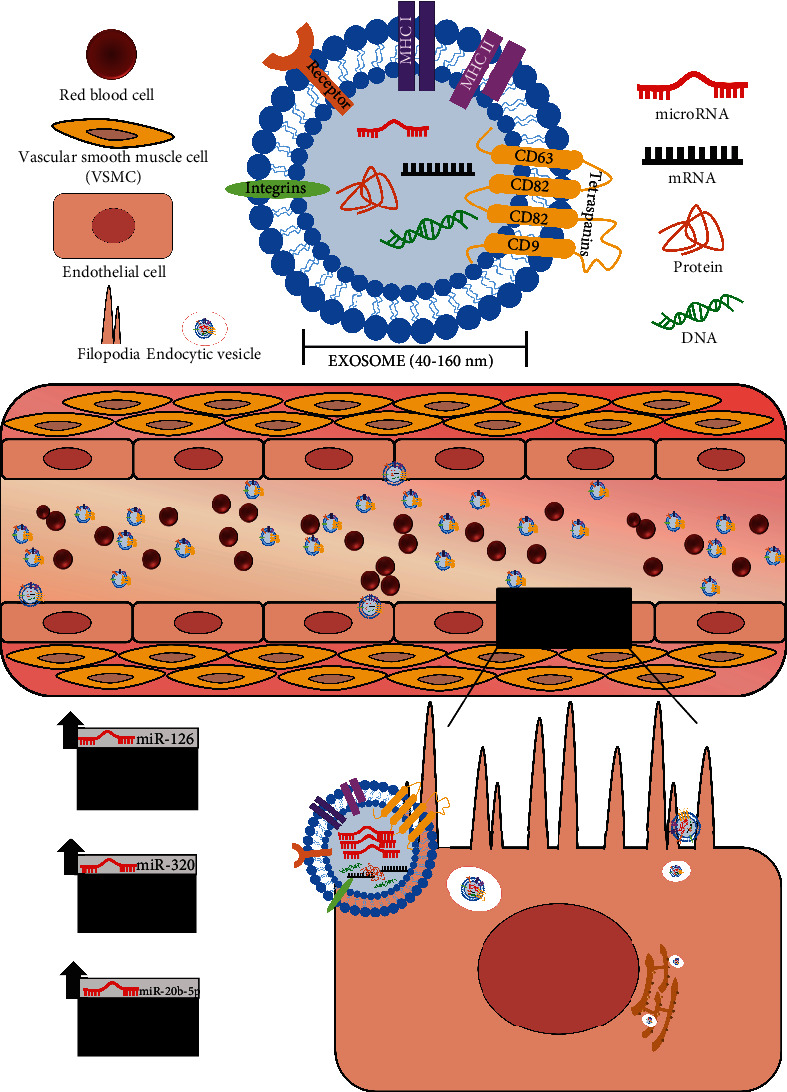
Exosomal miRNAs as mediators of endothelial dysfunction. Exosomes (40-160 nm) function in cell-to-cell communication via transfer of exosomal content (i.e., miRNAs, proteins, mRNA, and DNA) to neighboring or distant cells. Exosomes can be recruited to the endothelial cell body by filopodia surfing. They are then internalized and shuttled within endocytic vesicles and scan the endoplasmic reticulum and allow for entry of exosomal miRNA cargo into RNAi translation machinery to induce a functional response [62]. For example, miR-126 functions to improve vascular tone. However, a reduction in miR-126, as often reported in T2DM, worsens vascular tone and endothelial function. Exosomal enrichment of miR-320 and miR-20b-5p impairs endothelial proliferation and angiogenesis. Thus, the uptake of exosomes enriched with certain miRNAs can impair endothelial function and further accelerate the microvascular complications associated with type 2 diabetes mellitus.

In addition to exosomes serving as early markers of endothelial dysfunction in type 2 diabetes mellitus, previous research highlights the unique role of exosomes as mediators of disease [48, 63]. Exosomes and exosomal content are released from a parent cell and can be taken up by neighboring or distant cells to facilitate intercellular communication and transfer of its exosomal cargo (i.e., miRNAs), functioning like hormones (Figure 1) [48–50]. Briefly, exosomes can surf on cellular protrusions called filipodia at the surface of target cells [64]. The filopodial base is a known hotspot for endocytosis and can facilitate cell entry of enveloped viruses and bacteria. Similarly, exosomes are recruited to the cell body via filopodia, internalized, and shuttled within endocytic vesicles to scan the endoplasmic reticulum as a potential site of cargo release [64]. Thus, a directed transport of exosomes to the endoplasmic reticulum membrane would allow for efficient entry of exosomal miRNA cargo into RNAi translation machinery [64].

The endothelium is in constant contact with microparticles, such as exosomes, that can regulate hemostasis and vascular permeability [65]. Exosomes (via exosomal content) participate in the pathological process of endothelial dysfunction by transferring its encircled biological information to recipient cells [66]. While this process is not fully understood, it may involve four distinct processes including direct ligand-receptor interaction, microparticle internalization, fusion of microparticle and target-cell membranes, and transfer of surface receptors, proteins, mRNA, and noncoding RNAs (i.e., miRNAs and long noncoding RNAs) [65]. For example, serum exosomes can deliver arginase 1 protein to endothelial cells to inhibit NO production—inducing a functional response—during the development of diabetic endothelial dysfunction [48]. Recently, Wang et al. found that the protective effects of angiotensin-converting enzyme 2 (ACE2) on endothelial cell injury occur through the exosomal effects on mitochondrial function [67]. Another study by Liu et al. reports that endothelial microvesicle-mediated transfer of miR-92a-3p can regulate angiogenesis in recipient endothelial cells [68]. Here, the investigators found that atherosclerotic conditions promote the packaging of endothelial miR-92a-3p in endothelial microvesicles that can transfer functional miR-92a-3p to regulate angiogenesis in endothelial cells [68]. Although this study highlights the packing of miR-92a-3p in microvesicles (100 to 1000 nm) and not exosomes (40 to 160 nm), it provides insight into how exosomal may transport miRs in the regulation of vascular health. Bhattacharjee et al. also report how exosomes can mediate a functional response contributing to endothelial dysfunction [69]. Their investigation revealed that plasma exosomes from adults with severe obstructive sleep apnea/obesity hyperventilation syndrome (OSA/OHS) can induce endothelial dysfunction [69]. Similarly, Peng et al. found that extracellular vesicles from red blood cells under intermittent hypoxia impair endothelial function by impairing PI3K/Akt signaling, providing insight into the development of OSA-related hypertension [70]. In addition, previous work reports that high-shear stress or the shear-responsive transcription factor Kruppel-like factor 2 (KLF2) induces vascular endothelial cells to secret exosomes enriched with miR-143 and miR-145 [71]. These miRs subsequently regulate CAMk2d and ELK1 in smooth muscle cells, functioning to regulate quiescent versus proliferative phenotype of these cells [71]. Similarly, it was previously reported that exosomes containing miR-155 were secreted from KLF5 overexpression vascular smooth muscle cells [72]. These miR-155-rich exosomes were then taken up by endothelial cells, in which miR-155 inhibited proliferation and migration of endothelial cells and impaired endothelial barrier function [72]. In another study investigating the role of circulating myocardial miRs, researchers found that circulating exosomes and the exosomal cargo (i.e., myocardial miRs) were transferred to the bone marrow [73]. These transferred exosomal miRs were shown to then downregulate CXCR4 expression in bone marrow cells, mobilizing progenitor cells [73]. This work suggests that exosomes released from the ischemic heart can mediate a systemic response of bone marrow progenitor cells to the site of injury [73].

More specifically, several studies also report how exosomal miRNAs mediate functional responses in the setting of type 2 diabetes mellitus to impair endothelial function. For example, miR-126-enriched exosomes derived from mouse brain endothelial cells (EC-Exo) intravenously injected into T2DM mice after stroke were found to improve not only neurological and cognitive function but also increased vascular density and arterial diameter [53]. In addition, treatment with these miR-126-enriched exosomes increased endothelial capillary tube formation, suggesting the restorative effects of EC-exo treatment to regulate vascular changes of stroke in T2DM [53]. In a study investigating the angiogenesis in diabetic hearts, Wang et al. found an enrichment of miR-320 in exosomes isolated from Goto-Kakizaki rat cardiomyocytes and high glucose-treated cardiomyocytes [74]. Their work suggests that diabetic cardiomyocyte-derived exosomes are taken up or “absorbed” by endothelial cells, resulting in increased levels of miR-320, and facilitate the downregulation of IGF-1, Hsp20, and Ets2 [71]. In turn, the uptake of these exosomes by endothelial cells inhibits endothelial cell proliferation, migration, and tube-like formation via transfer of the antiangiogenic miR-320 [74]. Previous studies report an enrichment of exosomal miR-20b-5p in the T2DM patients [51]. Xiong et al. recently found that miR-20b-5p can slow both wound healing and angiogenesis [51]. In a series of in vivo and in vitro studies, they reveal how exosomes derived from the peripheral blood of T2DM patients impair wound healing. Mice with full-thickness cutaneous wounds on their backs were injected with exosomes isolated from plasma of both nondiabetic and T2DM patients [51]. Mice treated with exosomes from T2DM patients had a significantly slower wound closure rate [51]. They go on to show that exosomes enriched with miR-20b-5p from T2DM can transfer this miRNA into human umbilical vascular endothelial cells (HUVECs) to suppress the angiogenic effects by inhibiting Wnt9b/*ß*-catenin signaling pathway [51]. Recently, Hill and colleagues provided additional support for the role of extracellular vesicles (i.e., exosomes) in cell-cell cross-talk in podocyte dysfunction, which is common in diabetic nephropathy [75]. In this study, the investigators report the transfer of extracellular vesicles from glomeruli endothelial cells (GEnC) to podocytes to mediate podocyte function. They reported that podocytes treated with extracellular vesicles from activated GEnC (glucose and PAN treated) altered both mRNA and miRNA expression in these cells (podocytes) [75]. EVs from GEnCs caused increased mitochondrial stress in podocytes and an increased expression of podocyte miR-200c-3p, which resulted in decreased VEGF production [75]. Another study by Prattichizzo et al. provides evidence that CD31^+^ vesicles (i.e., vesicles specifically from endothelial cells, immune cells, or platelets) from T2DM patients can not only shuttle a discriminatory type 2 diabetic miRNA signature but can also promote proinflammatory pathways in endothelial cells to drive the endothelial dysfunction underlying the many vascular complications associated with type 2 diabetes [76]. Collectively, the findings of these studies provide support for the unique role of exosomal cargo in inducing a functional response in the pathophysiology of endothelial dysfunction by transferring intravesicular content, in particular miRNAs.

## 7. Exosomal miRNAs as Therapies for Endothelial Dysfunction

The use of exosomes as potential therapeutic agents is an exciting and rapidly evolving area of research. Although much of this work has been initiated in cancer research, it has significant implications for a number of complex diseases, including T2DM and its macro and microvascular complications. The rich diversity of proteins, lipids, mRNAs, and miRNAs encircled in exosomes not only makes them ideal markers for disease but also highlights the use of these naturally released nanovesicles as potential therapeutic agents. Early studies report that extracellular vesicles (i.e., exosomes) released from mesenchymal stem cells can mediate cytoprotective, angiogenic, and regenerative effects [77–80]. For example, extracellular vesicles released from endothelial progenitor cells can stimulate angiogenesis through transfer of miR-126 and miR-296 in the endothelial cell culture [80]. Xiao et al. also presented evidence that endothelial cell-derived exosomes can provide protection against ischemia/reperfusion injury, suggesting the use of these exosomes as a potential treatment for neurological damage during ischemia/reperfusion [79]. Another example of exosome-based therapy for disease is the use of mesenchymal stem cell- (MSC-) derived exosomes to treat stroke in animals [81, 82]. Xin et al. demonstrated that exosomes derived from MSC improve functional recovery after stroke in rats by improving neurologic outcome and neurovascular remodeling [81]. More specifically, Venkat et al. presented recent evidence highlighting the therapeutic potential of exosomes enriched with miR-126 to improve the effects of diabetic stroke [53]. Collectively, these studies suggest the use of exosomes as potential biotherapeutics.

In addition to the therapeutic potential of naturally released exosomes to mediate functional responses in disease development, exosomes are also an attractive tool for therapeutic delivery. Exosomes serve as vehicles for a range of biomolecules, providing a stable environment for it encircled content as it travels through circulation. Similarly, exosomes provide a stable environment for targeted and controlled release of therapeutic agents. Exosomes can be modified to improve cell homing, fuse with the plasma membrane of cells to provide a direct entry point into the cell, and decrease immune response (via allogenic exosomes) [83]. There are several approaches for loading exosomes with therapeutic cargo. These approaches include loading naïve exosomes isolated from parental cells ex vitro, transfecting, or infecting parental cells with DNA encoding therapeutically active compound that are later released in exosomes and loading parental cells with a specific drug that is released in exosomes [83, 84]. Sun et al. provide one of the first reports of the biotherapeutic potential of exosomes. In this study, the investigators reveal that exosomes can successfully carry and deliver the anti-inflammatory molecule curcumin, suggesting the additive power of the functional properties of this small compound and exosomes to improve a functional response (inflammation) [85]. Wang and colleagues also provide strong evidence for exosome-based therapeutics in diabetic wound healing [86]. By developing a hydrogel containing adipose-derived mesenchymal stem cell exosomes, the researchers improved diabetic wound healing, in part, by promoting angiogenesis and blood vessel formation [85]. If these findings hold true, then similar methodologies could be employed to improve vascular function in T2DM.

## 8. Conclusion

Exosomes are mediators of intercellular communication, providing an attractive means to understand the complex underpinnings of diabetes-associated endothelial dysfunction. In addition, exosomes provide a unique tool to identify early noninvasive markers of disease. Finally, exosomes have huge biotherapeutic potential, serving as carriers of therapeutic molecules. One challenge limiting the translation of exosomes is the lack of a standard and efficient isolation and characterization techniques. The isolation, quantification, and characterization of exosomes limit its translation from bench to bedside. Differential ultracentrifugation is currently the gold standard for isolating exosomes [87]. However, this process is time-consuming. Additional methods have been developed to circumvent challenges associated with ultracentrifugation, which include immunoprecipitation and size filtration [87]. However, the use of these methods typically results in a polluted mixture of extracellular vesicles. Despite this limitation, exosomes still hold significant promise as early and more sensitive markers of disease.

Exosomes and its content introduce an interesting advantage that provides a potential noninvasive “snapshot” or insider's view of what may occur during disease etiology. For example, in the microvascular complication DN, microalbuminuria (30-300 mg/day) has been used as a biomarker. However, Microalbumuniruai is a late feature of DN and manifests after significant and irreversible damage to the kidney [88]. In addition, the degree of proteinuria does not always reflect the severity or prognosis of DN, and not all diabetic patients with declining kidney function present with proteinuria [88]. Several colleagues have shown the potential prognostic and diagnostic power of exosome and exosomal content, which have been previously discussed. For instance, both Kumari et al. [60] and Mohan et al. [33] report the prognostic potential of urinary exosomal miR-451. Mohan et al. found that urinary exosomal miR-451 at 6 weeks predicted urine albumin at 9 weeks [33], supporting the use of exosomes as early and sensitive noninvasive markers of DN. Although the isolation of exosomes and exosomal content can be arduous, the wealth of information these vesicles hold makes for worthwhile studies. Exosomes and exosomal content can provide necessary insight into the early mechanisms of diabetes-induced vascular complications, mechanisms that may not currently be fully understood or identified. In addition, investigating exosomes introduces attractive avenues of therapeutic exploration. Additional work is needed to strengthen our understanding of exosomes and the translational utility of exosomal microRNAs as markers, mediators, and therapies for endothelial dysfunction in type 2 diabetes. As we look to the future, exosomes will play a vital role in our developing understanding of complex diseases and vascular complications like type 2 diabetes and its associated micro and macrovascular complications.

## Figures and Tables

**Table 1:** Extracellular miRNAs associated with type 2 diabetes mellitus and endothelial dysfunction.

miRNA	Normaglycemic	T2DM	Targets/pathways	Source	Complication	Reference
miR-126	Up	Down	SPRED-1 PIK3R2/p85- ß – PLK4	Plasma	Impaired angiogenesis	[20, 22, 89]
miR-26a	Up	Down	TRPC6	Plasma	Impaired angiogenesis	[90]
miR-133b	Down	Up	MAPK/ERK signaling	Serum	Diabetic nephropathy	[61, 91]
miR-342	Down	Up	SRY-box 6 (SOX-6)	Serum	Diabetic nephropathy	[61, 92]
miR-30a	Down	Up	TGF-*ß* 1 Becn1	Serum	Diabetic nephropathy	[61, 93]
miR-326		Up	ADIPOR-2 (adiponectin)	Plasma	T2DM	[40]
Let-7a	Down		Glucose metabolism	Plasma	T2DM	[40]
Let-7f	Down		Glucose metabolism	Plasma	T2DM	[40]
miR-20b-5p	Down	Up	AKT-interacting protein	Serum	T2DM	[52]
miR-21-5p	Down	Up	WWP1 (WW domain-containing protein 1) Endothelial progenitor cell proliferation	Plasma	T2DM	[94, 95]
miR-375-3p	Down	Up	*ß*-cell function	Serum	T2DM	[96]
miR-362-3p	Down		ADAMTS1	Plasma	Atherosclerosis (CAD)	[97]
miR-15a	Down	Up	UCP-2	RBC	T2DM	[6, 34]
miR-15b	Down	Up	TNF-alpha SOCS3	RBC	Pre-T2DM	[6]
miR-499	Down	Up	PTEN	RBC	Pre-T2DM	[6]
miR-7	Down	Up	mTOR signaling	Serum	Vascular complications	[98]
miR-25-3p		Up	CDH1 and PTEN	Plasma	Diabetic retinopathy	[36]
miR-320b	Down	Up	Angiogenesis	Plasma	Diabetic retinopathy	[36]
miR-495-3p	Down	Down		Plasma	Diabetic retinopathy	[36]
miR-34a	Down	Up	Sirt1	Mouse aortic endothelial cells	Endothelial dysfunction	[25]
miR-210	Down	Up	Cell proliferation	Serum	Diabetic retinopathy	[37]
miR-210	Up	Down	PTP1B	RBC	T2DM	[38]
miR-139-5p	Down	Up	c-Jun	Peripheral blood	T2DM	[35]

**Table 2:** Exosomal microRNAs associated with type 2 diabetes mellitus and endothelial dysfunction.

miRNA	Normaglycemic (concentration/excretion)	T2DM (concentration/excretion)	Targets/pathways	Source of exosomes	Method of isolation	Complication	Reference
miR-451	Low	High	YWHAZ CAB39	Urine	Ultracentrifugation	Diabetic nephropathy	[33, 54, 60]
Let-7c-5p	Low	High		Urine	Differential centrifugation	Diabetic nephropathy	[99]
miR-192	Low	High	Egr1	Urine	Ultracentrifugation	Diabetic nephropathy	[100, 101]
Let-7e-5p	Low	High	FASLG (migration and tube formation of endothelial progenitor cells	Urine	miRCURY™ exosome isolation kits (Qiagen)	Diabetic nephropathy	[58, 102, 103]
miR-15b	Low	High	TNF-alpha	Urine	Ultracentrifugation	Diabetic nephropathy	[59]
miR-34a	Low	High	Growth arrest specific 1 (GAS1)				
miR-636	Low	High	Adipogenesis				
miR-30b-5p	Low	High	Epithelial-to-mesenchymal transition	Urine	Ultracentrifugation	Diabetic nephropathy	[58]
miR-20-5p	Low	High	Wnt9b/*ß*-catenin signaling pathway	Peripheral blood	Ultracentrifugation	Wound healing Angiogenesis	[49, 50]
miR-126	Low	High	Angiogenesis Vascular integrity	Mouse brain endothelial cells	ExoQuick-TC (exosome precipitation solution kit, system biosciences)	Stroke	[53]
miR-320c	Low	High	ADAMTS5 CDK6 TSP-4 BMP6	Urine	ExoQuick-TC (exosome precipitation solution kit, system biosciences)	Diabetic nephropathy	[28]
Let-7i-3p	Low	High	Wnt/b-catenin signaling cascade, activin receptor signaling, and cell differentiation and proliferation	Urine	miRCURY™ exosome isolation kits (Qiagen)	Diabetic nephropathy	[62]
miR-24-3p	Low	High				Diabetic nephropathy	
miR-27b-3p	Low	High				Diabetic nephropathy	
miR-15b-5p	High	Low				Diabetic nephropathy	
miR-25-3p	Low	High	Endothelial cell proliferation Angiogenesis	Plasma	ExoQuick-TC (exosome precipitation solution kit, system biosciences	Diabetic retinopathy	[36]
miR-320b	Low	High					
miR-495-3p	Low	High					

## Data Availability

The data used to support the findings of this study are available from the corresponding author upon request.
